# *Morus alba* L. Plant: Bioactive Compounds and Potential as a Functional Food Ingredient

**DOI:** 10.3390/foods10030689

**Published:** 2021-03-23

**Authors:** Centhyea Chen, Umi Hartina Mohamad Razali, Fiffy Hanisdah Saikim, Azniza Mahyudin, Nor Qhairul Izzreen Mohd Noor

**Affiliations:** 1 Faculty of Food Science and Nutrition, Universiti Malaysia Sabah, Kota Kinabalu 88400, Sabah, Malaysia; centhyea97@gmail.com (C.C.); umi.hartina@ums.edu.my (U.H.M.R.); 2 Institute of Tropical Biology and Conservation, Universiti Malaysia Sabah, Kota Kinabalu 88400, Sabah, Malaysia; fiffy@ums.edu.my (F.H.S.); azniza@ums.edu.my (A.M.)

**Keywords:** *Morus alba*, bioactive compounds, functional food ingredients

## Abstract

*Morus alba* L. (*M. alba*) is a highly adaptable plant that is extensively incorporated in many traditional and Ayurveda medications. Various parts of the plant, such as leaves, fruits, and seeds, possess nutritional and medicinal value. *M. alba* has abundant phytochemicals, including phenolic acids, flavonoids, flavonols, anthocyanins, macronutrients, vitamins, minerals, and volatile aromatic compounds, indicating its excellent pharmacological abilities. *M. alba* also contains high nutraceutical values for protein, carbohydrates, fiber, organic acids, vitamins, and minerals, as well as a low lipid value. However, despite its excellent biological properties and nutritional value, *M. alba* has not been fully considered as a potential functional food ingredient. Therefore, this review reports on the nutrients and bioactive compounds available in *M. alba* leaves, fruit, and seeds; its nutraceutical properties, functional properties as an ingredient in foodstuffs, and a microencapsulation technique to enhance polyphenol stability. Finally, as scaling up to a bigger production plant is needed to accommodate industrial demand, the study and limitation on an *M. alba* upscaling process is reviewed.

## 1. Introduction

*Morus alba* Linn (*M. alba*), also known as white mulberry, belongs to the Moraceae family [1]. It is a small deciduous tree cultivated in various tropical, subtropical, and temperate countries, including China, Japan, Korea, Thailand, Indonesia, India, Vietnam, Brazil, Africa, and others [2,3]. Many traditional medicines incorporate *M. alba* fruit, leaves, roots, branches, and bark in Ayurveda medication systems due to their health benefits and antioxidants [4].

*M. alba* contains abundant bioactive compounds, including phenolic acids, flavonoids, flavonols, anthocyanins, macronutrients, vitamins, minerals, and volatile aromatic compounds [5,6]. Its fruits and leaves contain significant amounts of quercetin, rutin, and apigenin; ferulic, chlorogenic, and protocatechuic acids are also the significant compounds in fruits. These natural bioactive compounds hold potent biological activities proven to exhibit excellent pharmacological effects against various diseases. These include antioxidative, diuretic, antiobesity, hypoglycemic, hypotensive, anticholesterol, antidiabetic, and antimicrobial properties [7,8]. Moreover, the high quantity of phenolic compounds also contributes to *M. alba*’s functional properties in food applications. For example, the flavonoids and caffeoylquinic acids in *M. alba* could benefit as colorants, flavorants, food fortificants, antioxidants, preservatives, and antimicrobial agents against bacteria and fungi, all of which are essential in the food industry. Simultaneously, their anthocyanins could act as natural antioxidative food colorants [9,10].

That aside, *M. alba* as a whole also has a high nutraceutical value due to its low lipid value and high levels of protein, carbohydrates, fiber, organic acids, vitamins, and minerals that are comparable to other berries [6,11]. To be precise, *M. alba* contains approximately 3.6 g/100 g DW crude fiber and 19.4% protein content, giving it great potential in contributing to the recommended dietary allowance (RDA) of proteins [12]. Moreover, macroelements such as Ca, N, K, and Mg were abundantly found in both leaves and fruit, with low Na values of 0.01 g/100 g DW, making them suitable for low-sodium diets [11,12].

As a result of public and industry interest in the importance of functional foods in disease prevention, a rise in active food-market exploitation has occurred. With their demonstrated nutrition and health benefits, *M. alba* leaves and fruit can be considered as suitable ingredients to contribute to a broader application of functional foods. *M. alba* products can widely be found in the market, including mulberry powder, dried fruit, juices, jellies, jams, marmalade, ice cream, desserts, candies, pastes, and wine. However, its potential is limited in producing food products, and its efficiency as a functional food product still needs to be discovered. Therefore, there are still many possibilities to be explored, as it is imperative to sustain and preserve *M. alba* health benefits in food. This review paper aims to discuss the nutritional and phytochemical properties of *M. alba* leaves, fruit, and seeds and their potential as food ingredients for developing novel and functional foods to enrich human nutrition.

## 2. Nutrients and Phytochemical Compositions of *Morus alba*

### 2.1. Nutrient Composition of Morus alba

People consume mulberry species in various countries due to their nutritiousness, deliciousness, nontoxicity, and abundant active benefits. The leaves of *M. alba* species are rich in protein, carbohydrates, fiber, and vitamins, especially ascorbic acid and β-carotene [13]. Studies have also found that the leaves contain a high amount of important minerals such as calcium (Ca), potassium (K), magnesium (Mg), zinc (Zn), and many others. Moreover, according to Sánchez-Salcedo et al. [12], *M. alba* leaves possessed high iron (Fe) values (119.3–241.8 mg/kg) and a low level of sodium (0.01 mg/100 g), making them a suitable diet material for sodium-restricted individuals. The leaves also contain a considerable amount of organic acids, including citric acid (0.26–3.85 mg/g FW), malic acid (7.37–12.49 mg/g FW), tartaric acid (0.085–0.212 mg/g FW), succinic acid (1.02–5.67 mg/g FW), lactic acid (0.29–0.83 mg/g FW), fumaric acid (0.058–0.39 mg/g FW), and acetic acid (0.029–0.1 mg/g FW), which contribute to the potential health benefits of *M. alba* leaves [14].

The same nourishing richness is in *M. alba* fruit, with a protein content higher (10.15–13.33%) than other mulberry species [6]. A study by Owon et al. [15] showed a higher protein value of *M. alba* fruit (12.98%) as compared to black mulberry (10.85%), golden berry (9.16%), and strawberry (7.65%). Their great protein amount has proven their capability in contributing to protein’s recommended dietary allowance (RDA), which is 0.8 g/kg of body weight [16]. A considerable percentage of minerals, including N, P, K, Mg, Mn, Ca, Zn, Cu, Fe, and Se, were observed in *M. alba* fruits, while a higher ascorbic acid value was obtained from *M. alba* fruit (22.4 mg/100 g) than the *M. rubra* species (19.4 mg/100 g) [1,11]. A summary of these macro- and microelements can be seen in Table 1 and Table 2.

### 2.2. Phytochemicals of Morus alba Leaves

Flavonoids are essential bioactive compounds with excellent antioxidant properties that are found in *M. alba* leaves. Thabti et al. [24] reported the presence of three newly identified compounds (kaempferol-7-*O*-glucoside, quercetin-3-*O*-rhamnoside-7-*O*-glucoside, and quercetin 3-*O*-β-glucoside-7-*O*-α-rhamnoside), along with 10 other known compounds (1-caffeoylquinic acid, 5-caffeoylquinic acid, 4-caffeoylquinnic acid, caffeic acid, rutin, quercetin-3,7-d-*O*-β-d-glucopyranoside, quercetin-3-*O*-glucoside, quercetin-3-*O*-(6-malonyl)-β-d-glucopyranoside, kaempferol-3-*O*-glucopyranosyl-(1,6)-β-d-glucopyranoside, and kaempferol-3-*O*-(6-malonyl)glucoside). These findings were supported by Sánchez-Salcedo et al. [5] and Memon et al. [25]. They found chlorogenic, gallic, vanillic, p-hydroxybenzoic, syringic, p-coumaric, protocatechuic, ferulic, and m-coumaric acids as the leaves’ major phenolic acids.

Several new compounds, including flavan derivatives (moracinflavan A-G [26]) and 2-arylbenzofuran derivatives (moracinfurol A and B) were obtained from *M. alba* leaves [27]. The same significant phytochemical groups—alkaloids, phenolic, flavonoids, tannin, and terpenes—were isolated from leaves of two different maturities, with mature leaves containing higher 1-deoxynojirimycin (DNJ) and secondary metabolite values [28]. However, only simple terpenes were isolated via pyrolysis gas chromatography–mass spectrophotometry (Py/GC/MS), possibly due to terpenes’ lack of a polar group, which makes them more easily volatile compared to alkaloids and phenolics. Further analysis with different pyrolysis temperatures might isolate more compounds from *M. alba* leaves [29]. Ultimately, rutin, apigenin, and quercetin were the three highest bioactive constituents among the major phytochemical classes of *M. alba* leaves (Table 3) [30]. In addition, the presence of alkaloids, carbohydrates, fatty acids, phytosterols, glycosides, proteins, tannins, gums, and amino acids were also observed in *M. alba* leaves in five different solvents [31].

### 2.3. Phytochemicals of Morus alba Fruit

*M. alba* fruit is not commonly integrated into traditional medicine, possibly due to its seasonal production and the limited dissemination of its health benefits. However, the increasing interest in analyzing, isolating, and quantifying *M. alba* fruit phytochemicals has led to reports of their richer phenolic- and volatile-compound content, as well as better antioxidant capacity than other berry species like blueberry, strawberry, blackberry, and raspberry [38]. Previously, Wang et al. [39] found a total of 17 phenolic compounds in the fruits, consisting of cinnamic acid derivatives (0.36–1.29 mg/g DW), flavonols (0.07–0.36 mg/g DW), anthocyanins (not quantified), and benzoic acid derivatives (0.81–2.33 mg/g DW); while caffeoylquinic acids (CQAs) lower than in the leaves were detected (0.16–3.62 mg/g DW and 6.78–8.48 mg/g DW, respectively) [5].

Calín-Sánchez et al. [40] found 35 volatile compounds and categorized them into aldehydes, esters, ketones, benzene terpenes, and oxygenated terpenes. Moreover, the presence of bioactive compounds in *M. alba* fruit, including rutin (293.5 μg/g), chlorogenic acid (226.9 μg/g), caffeic acid (17.2 μg/g), quercetin (15.2 μg/g), gallic acid (8.9 μg/g), kaempferol (5.8 μg/g), and apigenin (3.5 μg/g), has previously been detected [41]. The fruit’s major fatty acids were also reported, including linoleic acid (57.26%), palmitic acid (22.42%), oleic acid (10.5%), stearic acids (4.27%), and myristic acid (0.98%) [1]. Meanwhile, in a more recent study by Xu et al. [42], three new compounds were isolated: (2S,2″S)-2,3,8,9-tetrahydro-5-hydroxy-8-(1-methylethenyl)-2-(4-hydroxyphenyl)-4H-furo2,3-h]-1-benzopyran-4-one; (1″R,2″R)-4-(2-formyl-1H-pyrrol-1-yl)-1-(2-hydroxy-1-methylpropoxy)-butanoate; and (4′S,5′R)-1-[(Tetrahydro-4-hydroxyl-5-oxo-2-furanyl)methyl]-2,4(1H,3H)-pyrimidinedione.

Zhang et al. [43] suggested flavonols, flavonoids, anthocyanins, hydroxynamic acids, and benzoic acids as the significant polyphenol composition in *M. alba* fruit. The fruit’s polyphenol composition was significantly influenced by their maturity stages. The brackish fully ripe *M. alba* fruit extract contained higher total sugar and anthocyanins, while the ripe red fruit contained higher ß-carotene and ascorbic acid [44]. This is because sugar acts as a precursor in synthesizing anthocyanins, hence their higher value in mature fruit. A study by El-Baz et al. [45] found that types of solvent influenced the extraction of phytochemicals. Ethanol (EtOH) extract was the most efficient solvent, as 18 compounds were isolated, mostly esters (96.32%), followed by dichloromethane (DCM) and ethyl acetate (EtOAC) fractions with a total of 12 compounds. The major bioactive compound groups in *M. alba* fruit are shown in Table 4. The rich reports on *M. alba* fruit’s bioactive-compound content suggest its excellent potential for functional food-product development.

### 2.4. Phytochemicals of Morus alba Seeds

*M. alba* seeds alone are barely researched, as they usually are incorporated in the fruit analysis. The seeds are tiny in size and difficult to separate. However, the content of polyphenol in the seeds alone is currently becoming an interest to be explored. Lee et al. [48] have found and quantified 11 polyphenolic compounds in defatted *M. alba* seeds. These compounds are rutin (31.1–60.0 mg/100 g), 4-prenylmoracin (10.5–43.3 mg/100 g), quercitrin (7.2–34.2 mg/100 g), (+)-dihydroquercetin (13.2–33.1 mg/100 g), quercetin (15.8–19.5 mg/100 g), isoquercitrin (5.8–15.4 mg/100 g), chlorogenic acid (0.0–15.3 mg/100 g), moracin (4.7–7.2 mg/100 g), procatechuic acid (0.0–11.6 mg/100 g), and (+)-dihydrokaempferol and trans-resveratrol (<0.1 mg/100 g). Meanwhile, caffeic acid (1.66 mg/g), 3,4-dihydroxybenzoic acid (1.57 mg/g), rutin (1.54 mg/g), and cyaniding-3-rutinoside (1.30 mg/g) were the major compounds in *M. alba* seeds [49].

Studies have shown that *M. alba* fruit seeds were rich in carbohydrates, fatty acids, and protein [50,51,52]. Yılmaz and Durmaz [53] have successfully extracted oil amounting to 232–332 mg/g from *M. alba* seed, while lower contents of 220–280 mg/g and 213.3 mg/g were extracted by Yao et al. [54] and Argon et al. [50], respectively. Researchers have claimed that the seed oil contains high amounts of carbohydrates and protein, approximately 38–54.8% and 15–21.6%, respectively [50]. A high total tocopherol content of about 2031.20–2393.63 mg/kg, mostly comprising δ-tocopherol (1644.70–2012.27 mg/kg), γ-tocopherol (349.74–366.80 mg/kg oil), β-tocopherol (13.11–19.27 mg/kg oil), and α-tocopherol (6.60–12.35 mg/kg oil) were reported [50,53]. High antioxidative total phenolic content and total flavonoid content were also found in the seed oil. Moreover, the oil could be fractionized into mono-, di-, and triglycerides (1.47–2.05%, 3.25–4.10%, and 90.08–91.05%, respectively). The total lipids could be fractionized into the three major lipids groups: neutral lipids (92.97–93.10%), glycolipids (2.12–2.43%) and phosphor-lipids (1.85–1.92%) [55]. Interestingly, *M. alba* seed oil was reported to be rich in essential fatty acids, including linoleic acid (75.83–81.1%), palmitic acid (7.96–9.51%), oleic acid (5.64–10.38%), stearic acid (3.45–5.01%), linolenic acid (0.41–80.6%), and myristic acid (0.07%) [50,53]. Based on the capacity of *M. alba* seeds in oil form, it has a high potential, especially as a fat substitute in food and nonfood applications.

## 3. Nutraceutical Properties of *Morus alba*

### 3.1. Antioxidative Activity

*M. alba* is rich in bioactive compounds, and its flavonoids contain high antioxidative abilities, based on the analysis of different assays: 1,1-diphenyl-2-picrylhydrazyl (DPPH), 2,2-azinobis-(3-ethylbenzothiazoline-6-sulfonate) (ABTS), and ferric reducing antioxidant power (FRAP) (Table 5) [56]. The antioxidative property of flavonoids inhibited the oxidative modification of human lipoproteins [57]. Jha et al. [58] isolated 0.5–3 kDa of oligopeptides from *M. alba* leaves that possessed high scavenging activity in DPPH (322.67–876.92 μg/mL), ABTS (141.29–256.59 μg/mL), nitric oxide (5.11–176.38 μg/mL), and FRAP (24.32–258.99 AAE/g). The isolated peptides showed values of 169.55–328.57 μg/mL in ferrous ion chelation and 202.3–315.5 μg/mL in malondialdehyde (MDA) inhibition, which is important in hindering the production of highly reactive hydroxyl radicals.

*M. alba* fruit also has potent antioxidative activities. According to D’urso et al. [59], phenylpropanoids and flavonols were the two main compounds responsible for the fruit’s antioxidant activity. High total flavonoid content (187.23 mg/g QUE of DW) expressing strong DPPH (IC_50_ = 0.518 mg/mL) and FRAP (0.685 at 4.0 mg/mL) were obtained from *M. alba* fruit [34]. The flavonoids showed haemolytic and antihaemolytic ability in red-blood-cell haemolysis of H_2_O_2_ induced in mice. In addition, the inhibition of lipid peroxidation in the liver, microsome, and mitochondria were detected as 45.5%, 42.8%, and 39.4%, respectively. The total antioxidant activity of *M. alba* fruit was similar to that of strawberry (39.40 and 51.31 mg Trolox/g of fresh weight, respectively) [59].

**Figure 1:**
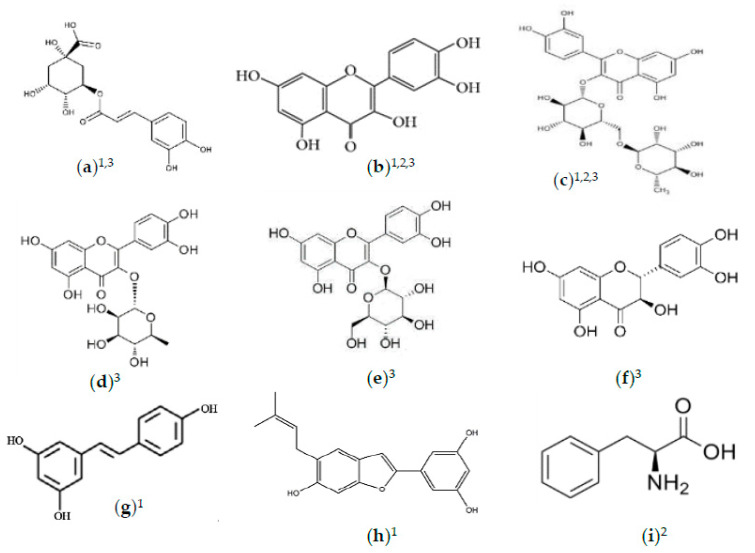
Natural compounds in *M. alba* with antioxidative abilities. (**a**) Chlorogenic acid; (**b**) quercetin; (**c**) rutin; (**d**) quercitrin; (**e**) isoquercitrin; (**f**) dihydroquercetin; (**g**) resveratrol; (**h**) moracin; (**i**) phenylpropanoids. ^1^ Refers to compounds in *M. alba* leaves; ^2^ refers to compounds in *M. alba* fruit; ^3^ refers to compounds in *M. alba* seeds.

Meanwhile, in *M. alba* seeds, stronger DPPH radical activity than L-ascorbic acid (IC_50_ = 31.5 μM) and α-tocopherol (IC_50_ = 52.3 μM) were revealed by rutin (IC_50_ = 20.2 μM), isoquercitrin (IC_50_ = 22.5 μM), quercitrin (IC_50_ = 24.6 μM), quercetin (IC_50_ = 27.8 μM), (+)-dihydroquercetin (IC_50_ = 28.9 μM), and chlorogenic acid (IC_50_ = 30.6 μM) [48]. These results indicate that *M. alba* contains antioxidative phenolic compounds potentially useful as nutraceutical agents in functional foods (Figure 1). However, temperature influences the contents of phenolic acids and flavonoids during drying processes [60]. Therefore, studies using different drying methods, type of solvents, and extracting conditions that are more efficient and can effectively maintain yields, bioactive components, and biological activities should be pursued. Overall, the existing literature has evidently indicated the efficient antioxidative capacity of *M. alba* both in vivo and in vitro, making *M. alba* a promising ingredient for nutraceutical development.

### 3.2. Antidiabetic Property

Natural plant extracts can ameliorate insulin production and inhibit intestinal glucose absorption, thus becoming a significant catalyst in managing diabetes [63]. Ahn et al. [64] supplemented *M. alba* in diabetic mice for 14 days and found a significant reduction of plasma total cholesterol, hepatic T-C, and triglyceride concentrations. However, the *M. alba* leaf-supplemented group showed the most significant activities, including decreased plasma glucose and insulin, and elevated levels of protein S6 kinase (pS6K), phosphorylated Akt (pAKT), and phosphorylated (p)-AMP-activated protein kinase (pAMPK). These showed that *M. alba* leaves have better antidiabetic and antidyslipidemic properties compared to the fruits. Moreover, *M. alba* leaf extract and oligopeptides (0.5–3 kDa) were found to create potent inhibition of both α-glucosidase and α-amylase enzymes [58,65]. This allows a delay in the breakdown of sugars, thus reducing glucose’s absorption rate and controlling postprandial hyperglycemia. From this, it was discovered that *M. alba* leaves contain 30–170 mg/100 g DW of DNJ, which is a potent α-glucosidase inhibitor that effectively suppresses postprandial blood glucose elevation with just 6.5 mg of its administration [66]. Additionally, Jeon and Choi [67] have isolated eight compounds containing α-glucosidase inhibitory activity, with chalcomoracin (IC_50_ = 6.00 μM) and 4′-prenyloxyresveratrol (IC_50_ = 28.04 μM) holding the most significant inhibition ability.

That aside, a two-week supplementation of *M. alba* fruit extract could improve insulin sensitivity and reduce hepatic glucose production in type 2 diabetic mice [68]. The fruit-supplemented mice showed a significantly lower level of blood glucose and blood glycosylated haemoglobin (HbA1c) (9.083%) as compared to the diabetic-control and rosiglitazone mice groups (12.921% and 7.454%, respectively). The fruit extract also significantly enhanced the activation of pAMPK and p-Akt substrate of 160 kDa (pAS160). Consequently, it significantly increased the GLUT4 level in skeletal muscles and decreased the glucose 6-phosphatase and phosphoenolpyruvate carboxykinase levels in the liver, thus suppressing the hepatic gluconeogenesis process [68].

**Figure 2:**
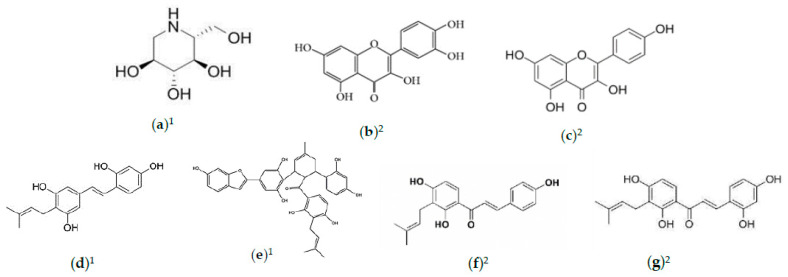
Natural compounds with antidiabetic activities. (**a**) 1-Deoxynojirimycin (DNJ); (**b**) quercetin; (**c**) kaempferol; (**d**) 4′-prenyloxyresveratrol; (**e**) chalcomoracin; (**f**) isobavachalcone; (**g**) morachalcone. ^1^ Refers to compounds in *M. alba* leaves; ^2^ refers to compounds in *M. alba* fruit.

A recent study reported 16 flavonoids from *M. alba* fruit possessing α-glucosidase-inhibitory ability [42]. Among them, quercetin (IC_50_ = 16.00 μM), kaempferol (27.61 μM), morachalcone (49.07 μM), and isobavachalcone (51.84 μM) were predominant, while the rest presented higher IC_50_ values than acarbose (122.22 μM). The structures of these compounds are shown in Figure 2. The inhibitory ability of flavonoids is interchangeable based on any slight structural changes from the hydroxylation of the C-3 position of dihydroflavones, which significantly increased the α-glucosidase-inhibitory ability of (2R)-eriodictyol (IC_50_ = 168.72 μM) as compared to (2R, 3R)-dihydroquercetin (IC_50_ = 348.12 μM) [42]. Overall, *M. alba* leaves and fruit contain high α-glucosidase-inhibiting, antihyperglycemic, and antidyslipidemic properties, making them a positive source for diabetes treatment.

### 3.3. Antihyperlipidaemia and Antiobesity Activity

Studies have found that the *M. alba* leaves could significantly reduce the levels of cholesterol, triglyceride, and lipid peroxidation in blood plasma and post a mitochondrial fraction of cholesterol-induced mice, followed by decreases in body-weight gain, the atherogenic index, coronary artery indices (CRIs), Lee’s index, and waist circumference [69,70]. The downregulation of leptin (0.39-fold) and resistin (0.71-fold), along with the upregulation of adiponectin (1.53-fold), have aided *M. alba* leaves to work directly on visceral fat mass and attenuated their adiposity and derangements. The abilities of *M. alba* leaves in suppressing obesity, reducing visceral adiposity, and cardiometabolic alteration was accredited to their polyphenols, particularly chlorogenic acid, quercetin, caffeic acid, rutin, and kaempferol [70].

**Figure 3:**
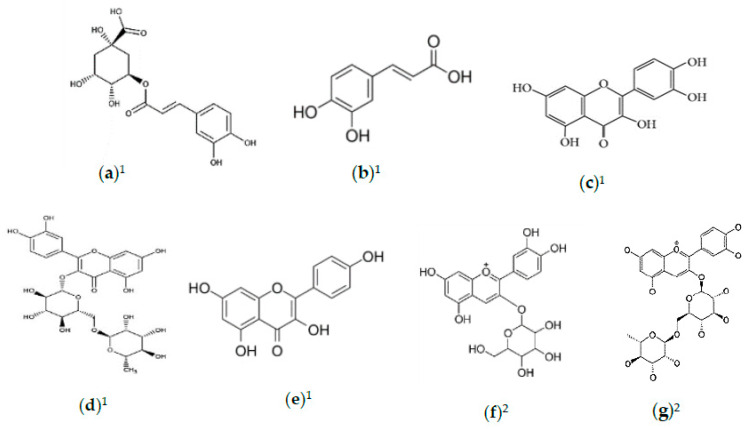
Natural compounds with antihyperlipidaemia activities. (**a**) Chlorogenic acid; (**b**) caffeic acid; (**c**) quercetin; (**d**) rutin; (**e**) kaempferol; (**f**) cyanidin-3-O-glucoside; (**g**) cyanidin-3-O-rutinoside. ^1^ Refers to compounds in *M. alba* leaves; ^2^ refers to compounds in *M. alba* fruit.

Ten compounds were found to have antiadipogenic activity, in which three were benzofuran derivatives, two were phenolic derivatives, two were flavonoids, one was an alkaloid, one was lignin, and one was coumarin. These compounds possessed 2.1–36.6% of antiadipogenic activity on 3T3-L1 adipocytes [71]. The efficacy of *M. alba* leaves to significantly reduce the expression of the key regulator of LDL receptor, the proprotein convertase subtilisin/kexin type 9 (PCSK9), was reported by Lupo et al. [72]. This report was followed with significant reduction of 3-hydroxy-3-methyl-3-glutaryl coenzyme A reductase (HMGCR) and fatty acid synthase (FAS) in mRNA levels of HepG2 cells by 51.1% and 37.2%, respectively. Whereas, at the protein level, expression of the LDL receptor was elevated by 1.8-fold. However, in hepatic cells, no significant effect on the PCSK9 promoter activity was found, despite its expression inhibition in both mRNA and protein levels.Both 5% and 10% *M. alba* fruit supplementation have significantly down-regulated serum and liver thiobarbituric-acid-related substances (TBARS) in mice [73]. In contrast, a significant increment of red blood cells, liver superoxide dismutase, and blood glutathione peroxidase levels only occurred in 10% fruit-supplemented mice. The fluctuated HDL-C and LDL-C in high-fat animals were claimed to be contributed by the total anthocyanin (0.0087%) and flavonoid (0.39%) contents in the fruit [73]. These findings showed the capacity of *M. alba* fruit to manage hyperlipidaemia. In another study, *M. alba* fruit modulated obesity-induced cardiac dysfunction by inhibiting lipogenesis and fibrosis, and enhancing lipolysis in high-fat diet-induced obesity [74]. Fruit extract also attenuated reactive oxygen species (ROS), vascular function, inflammatory markers, and lipid accumulation while reducing collagen in obese rats; these are considered factors for defense against and remediation of obesity-related cardiac dysfunction cardiac fibrosis. The chemical structures of compounds possessing antihyperlipidaemia can be seen in Figure 3. To sum up, *M. alba* leaf and fruit extracts contain significant obesity-repressing and cholesterol-reducing properties, making them an excellent defensive agent against obesity, atherosclerosis, and hyperlipidaemia-related disorder. Nevertheless, the active components’ mechanism to induce the uptake of LDLR and LDL is still unknown. Therefore, further study needs to explore a more profound understanding of *M. alba*’s antihyperlipidaemia mechanism.

### 3.4. Neuroprotective Ability

According to Gupta et al. [75] and Rayam et al. [76], *M. alba* leaves could facilitate gamma-aminobutyric acid (GABA) transmission, thus exhibiting an anticonvulsant ability in rats [77]. A 25 mg/kg methanolic leaf extract actually postponed the onset of pentylenetetrazole (PTZ)-induced chronic seizure. In contrast, 50 and 100 mg/kg extracts decreased convulsion time length [75]. These concentrations also significantly reduced the maximal electroshock-induced tonic hindlimb extension [75]. Meanwhile, *M. alba* leaves’ significant antiepileptic activity at 200 and 400 mg/kg at 6.8 and 3.16 s, respectively, was observed by Rayam et al. [76].

In the context of Alzheimer’s disease, a 28 day administration of 100 mg/kg ethanolic *M. alba* leaves enhanced both learning and memory function in spatial-memory-impaired rats, whereas the 200 mg/kg and 400 mg/kg extracts inhibited memory impairment [78]. A high dose (160 mg/kg daily) administration of DNJ could improve cognitive impairment, alleviate Aβ deposition in the hippocampus through β-secretase 1 (BACE1) inhibition, and reduce the expression of brain inflammatory factors such as TNF-α, IL1β, and IL-6 in mice [79]. DNJ minimizes the decline of brain-derived neurotrophic factor/tyrosine kinase receptor (BDNF/TrkB) signal pathway in mouse hippocampus. This therefore implies its ability in improving the hippocampal neuron microenvironment [79]. Similar results for *M. alba* fruit extract, including improved spatial memory and learning aptitude and decreased neuron apoptosis and Aβ plaques in mice hippocampus and cortex tissues were reported [80]. *M. alba* fruit also increased anti-inflammatory cytokines (IL-4) and reduced astrocytes and proinflammatory cytokines (TNF-α, IL-1β, and IL-6) in both hippocampus and cortex. Thus, it alleviated neuroinflammation [80]. That aside, *M. alba* fruit extract has significantly reversed the AlCl_3_ neurotoxin-induced alteration of striatum neurotransmitters and decreased 50% of acetylcholinesterase (AChE) activity in the brain. A significant increment in norepinephrine, epinephrine, 5-hydroxytryptamine serotonin, and dopamine neurotransmitter were observed in the treated mice [81]. Based on these data, *M. alba* could attenuate Alzheimer’s disease via Aβ deposition alleviation, inflammatory-factor reduction, and BDNF/TrkB signaling pathway alleviation, thus proving their protective effects on memory and learning abilities.

Parkinson’s disease is a dysfunctional motor disorder caused by progressive degeneration of dopaminergic neurons and glyphosate linked to oxidative stress [82]. According to Rebai et al. [82], *M. alba* leaf extract’s protective ability could fight the neurotoxicity and harmful effects of glyphosate. At the same time, it attenuated the levels of lactate dehydrogenase, malondialdehyde, and protein carbonyls. Leaf extract also chelated free ions to scavenge H_2_O_2_, and increased calcium and superoxide dismutase activity in the brain. These neuroprotective effects of *M. alba* leaves are due to synergism or antagonism among the bioactive phenolic components in the leaf extract.

**Figure 4:**
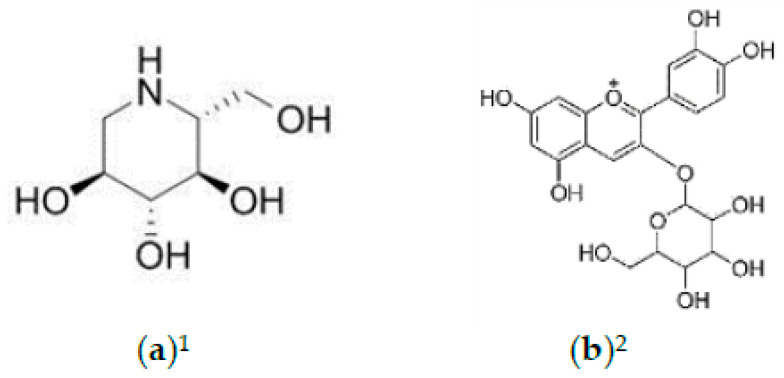
Structures of (**a**) 1-deoxynojirimycin (DNJ) and (**b**) cyanidin-3-O-glucoside. ^1^ Refers to compounds in *M. alba* leaves; ^2^ refers to compounds in *M. alba* fruit.

Moreover, *M. alba* fruit extract also significantly improved and delayed the progression of Parkinson’s-disease-like motor and nonmotor impairment symptoms in the 1-methyl-4-phenyl 1,2,3,6-tetrahydropyridine (MPTP)/probenecid-rat group [83]. *M. alba* fruit has inhibited olfactory dysfunction of rats based on shortened pellet retrieval time; amelioration of hypokinesia and bradykinesia, and inhibition of Gait dysfunction. Fruit extract also inhibited dopaminergic neuron degeneration and Lewy body formation through α-synuclein suppression, while amending the negative effects of MPTP/probenecid on ubiquitin expression in both the substantia nigra and striatum [83]. The cyanidin-3-glucoside (C3G) (Figure 4) from *M. alba* fruit has dose-dependently prevented membrane damage, and conserved mitochondrial function and mitochondrial membrane potential (MMP) of the primary cortical neurons in rats when exposed to oxygen–glucose deprivation for 3.5 h [84], suggesting the C3G neuroprotective effect. To sum up, results from various studies served as proof of the worthy neuroprotective effects of *M. alba* leaves and fruit. This makes them a promising nutraceutical ingredient to target neurodegenerative disorders, especially Alzheimer’s and Parkinson’s diseases.

### 3.5. Antimicrobial and Antiviral Activity

Over the past decade, the interest in plant-derived antimicrobial drugs has increased dramatically. Due to the high production cost and side effects of antibiotics, as well as their non-targeted mechanism of actions, various medicinal plants have been tested and showed positive effects on bacteria and microorganisms. Therefore, they are being used as a means against bacteria-caused diseases. Accordingly, *M. alba* leaves were reported to inhibit the growth of *Staphylococcus aureus*, *Bacillus cereus*, and *Pseudomonas fluorescence* [85]. However, out of the chosen 13 cultivars, only two cultivars showed low to moderate inhibitory effects on *Escherichia coli*. The extracts with the highest total phenolic contents could inhibit the three bacterial strains, verifying the correlation between the leaf extract’s total phenolic content and their antibacterial ability [85]. Thabti et al. [86] found that the aqueous and methanolic *M. alba* leaf extract showed antibacterial activity against *Salmonella* ser. *Typhimurium*, *Staphylococcus epidermis*, and *Staphylococcus aureus*.

With the increasing antibiotic-resistant microbial strains, De Oliveira et al. [87] determined the effect of ethanolic *M. alba* leaves on medical-related bacteria and fungi. In their study, the strains *Candida albicans* LM-106, *Candida albicans* ATCC-76645, and *Candida krusei* LM-656 developed resistance to the positive controls. Nevertheless, *M. alba* leaves have exerted a vigorous antimicrobial activity on *Candida albicans* LM-106 with a minimal inhibitory concentration of 256 μg/mL. Moderate activity was exerted on other strains: *Staphylococcus aureus* (ATCC-13150 and M-177), *Pseudomonas aeruginosa* (ATCC-9027 and P-03), *Candida albicans* (ATCC-76645 and LM-106), *Candida tropicalis* (ATCC-13803 and LM-6), *Candida krusei* (LM-656 and LM-978), and *Aspergillus flavus* (LM-714). *Staphylococcus aureus* showed the highest sensitivity to the leaf extract, while no effect against *Aspergillus niger* was observed.

**Figure 5:**
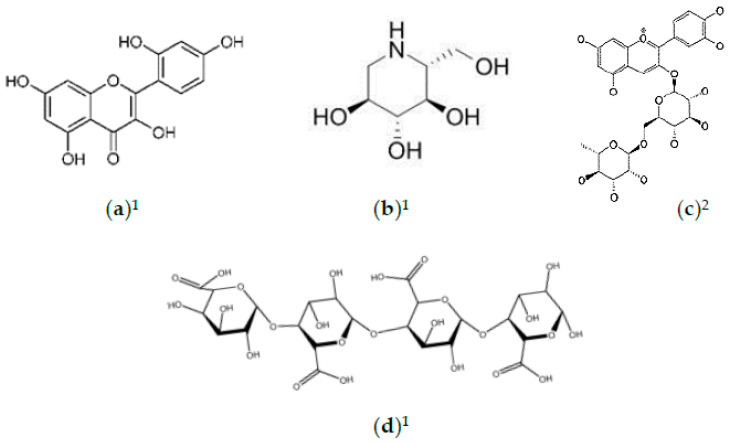
Natural compounds in *M. alba* possessing antimicrobial and antiviral activities. (**a**) Morin; (**b**) 1-deoxynojirimycin (DNJ); (**c**) cyanidin-3-O-rutinoside; (**d**) pectin.^1^ Refers to compounds in *M. alba* fruit; ^2^ refers to compounds in *M. alba* seeds.

On the other hand, morin isolated from *M. alba* fruit exerted moderate growth-inhibiting activity against *Streptococcus* spp. [88]. Investigation of the antimicrobial abilities of pectin isolated from *M. alba* fruit (Figure 5) showed antibacterial activity against selected Gram-positive and Gram-negative bacteria: *Bacillus cereus*, *Staphylococcus aureus*, *Streptococcus mutans*, *E. coli*, *Pseudomonas aeruginosa*, and *S.* Typhimurium at concentrations of 500–1000 µg/mL [89]. In addition, methanolic and water extracts of *M. alba* leaves were reported to be active against *S.* Typhimurium and *Staphylococcus aureus* [90]. However, acetone extract expressed better inhibitory activity on Gram-positive bacteria, followed by ethanolic and methanolic extracts. In contrast, no inhibition was detected in any of the extracts against Gram-negative bacteria [91]. In another study, an attempt at biotransforming *M. alba* fruit extract with *Lactobacillus brevis* DF01 and *Pediococcus acidilactici* K10 yielded effective antibacterial activity against *S.* Typhimurium through the reduction of growth and bacterial-produced biofilm [92]. Moreover, in Jacob et al. [93], DNJ derived from *M. alba* fruit was shown to exert a positive effect against bovine viral diarrhea virus (BVDV), GB virus-B (GBV-B), woodchuck hepatitis virus (WHV), and hepatitis B virus (HBV).

Meanwhile, *M. alba* seeds exerted an antiviral effect against the foodborne viral surrogates feline calicivirus-F9 (FCV-F9) and murine norovirus1 (MNV-1) [49]. The addition of 1 mg/mL of seed extract during pretreatment significantly inhibited FCV-F9 and MNV-1 plaque formation by 65% and 47%, respectively. Among the polyphenols, cyanidin-3-rutinoside showed the best reduction of MNV-1 polymerase gene expression. The significant inhibition of viral infection by *M. alba* seeds in the pretreatment stage suggested their best effect was on the initial stage of viral replication.

Based on these studies, the antimicrobial and antibacterial activity of *M. alba* leaves and fruit have been demonstrated to be effective, especially against *S.* Typhimurium, which is a globally known foodborne enteric pathogen accountable for acute gastroenteritis associated with undercooked or contaminated foods. Moreover, a lack of antiviral drugs could allow *M. alba* to be utilized as an effective alternative to prevent and control virus-related diseases. Nonetheless, further studies focusing on specific high antioxidative compounds and biotransformation are still required to support the antimicrobial and antiviral ability of *M. alba* to better control foodborne bacterial and viral surrogates.

### 3.6. Cytotoxicity and Anticancer Activities

Mulberry species are well known as a source of compounds that inhibit the initiation and development of cancers. The primary anticancer protective ability is due to the number of antioxidants available in *M. alba*. Several studies on various human cancer cells, including cervical cancer, lung carcinoma, hepatocellular carcinoma, breast cancer, and colorectal cancer have been conducted to verify the anticancer ability of *M. alba*. A study on methanolic *M. alba* leaf extract used against P19 embryonal carcinoma showed the highest cytotoxic effect with IC_50_ of 273, 117, and 127 μg/mL at 48 h, 96 h, and 144 h of treatment, respectively, as compared to the other 3 plants extracts [94]. In Fathy et al. [95], leaf extract inhibited HepG2 proliferation by repressing nuclear factor kappa B (NF-κB) gene expression and modulating the biochemical markers alfa-fetoprotein (AFP), alkaline phosphatase (ALP), gammaglutamyl transpeptidase (γ-GT), and albumin (ALB). Moreover, the leaves induced morphological changes in HepG2 cells to a more mature hepatocyte form [95]. Morphological changes also occurred in HeLa cells with 50 mM of morin isolated from *M. alba* leaves, followed by the formation of flocculent apoptosomes at 150 mM and spherical suspensions of dead/apoptotic cells at 220 mM. This morin-induced apoptosis occurred via multiple pathways, including increment of death-receptor expression, mitochondrial pathway, the elevation of apoptosis-related gene expression, and ROS-induced apoptosis [96].

**Figure 6:**
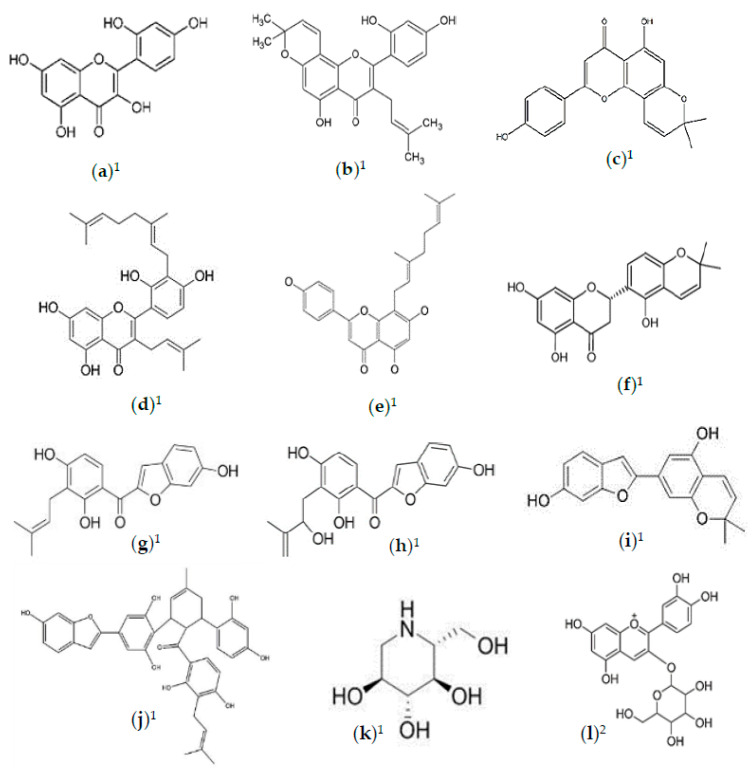
Natural compounds in *M. alba* with anticancer abilities. (**a**) Morin; (**b**) morusin; (**c**) atalantoflavone; (**d**) 3′-geranyl-3-prenyl-2′,4′,5,7-tetrahydroxyflavone; (**e**) 8-geranylapigenin; (**f**) sanggenon K; (**g**) morachalcone B; (**h**) morachalcone C; (**i**) moracin D; (**j**) chalcomoracin; (**k**) 1-deoxynojirimycin (DNJ); (**l**) cyanidin-3-glucoside. ^1^ Refers to compounds in *M. alba* leaves; ^2^ refers to compounds in *M. alba* fruit.

Interestingly, Fallah et al. [97] observed that flavonoids from *M. alba* leaves could induce a more potent dose-dependent cytotoxicity in colon cancer, compared to the established chemotherapy drug doxorubicin or their combination (flavonoids + doxorubicin). Dat et al. [2] found 10 flavonoid compounds exerting potent cytotoxicity toward HeLa cells (IC_50_ of 0.64–3.69 μM), MCF-7 (IC_50_ of 3.21–7.88 μM), and Hep-3B (IC_50_ of 3.09–9.21 μM). As such, the top three flavonoids with the highest cytotoxicity for each cancer cell were morusin, atalantoflavone, and 3′-Geranyl-3-prenyl-2′,4′,5,7-tetrahydroxyflavone in HeLa cells; 8-Geranylapigenin, sanggenon K, and 3′-Geranyl-3-prenyl-2′,4′,5,7-tetrahydroxyflavone in MCF-7 cells; and sanggenon K, 8-Geranylapigenin, and atalantoflavone in Hep-3B cells. Meanwhile, a more recent study suggested *M. alba* leaves had in vitro and in vivo cytotoxic activity on a number of compounds, including morusin, morachalcone B, morachalcone C, moracin D, moracin X, chalcomoracin, and DNJ [32]. The chemical structures of these compounds are shown in Figure 6.

Furthermore, in the area of different extraction solvents, n-hexane fractions of *M. alba* fruit expressed the best cytotoxicity on HCT116 (IC_50_ = 32.3 µg/mL), while dichloromethane (DCM) fractions expressed best on MCF7 (IC_50_ = 43.9 µg/mL) [45]. Moreover, the DCM fruit fraction was safely recognized based on its zero effect on a normal cell line (Bj1), while EtOAc fraction afflicted 47.3% cytotoxicity on Bj1. Nonetheless, low to no effect was seen from the extracts on HepG2 (0–22.8%) and PC3 cancer cells (0–39.6%). This showed that a sample’s cytotoxicity varied with extraction yields based on the solvent’s varying polarity [98]. Cho et al. [99] showed that the cyanidin-3-glucoside (C3G) from *M. alba* fruit could inflict cytotoxicity and dose-dependently increased human breast cancer cell death. *M. alba* C3G’s active apoptosis occurred via elevation of cleaved caspase-3, reduction of Bcl-2, and DNA fragmentation. Moreover, 25 days of a C3G diet dose-dependently reduced the size of the tumor in tumor-transplanted mice. This proves their capability to inhibit the proliferation and growth of cancer cells in both in vitro and in vivo models [99].

A newly found indole acetic acid derivative from *M. alba* fruit revealed dose-dependent cytotoxicity on HeLa cells. The apoptosis mechanism was deduced via the death-receptor-mediated extrinsic pathway and mitochondria-mediated intrinsic path owing to the activation of caspase-8 and -9 [100]. Intriguingly, Ramis et al. [101] observed that the leaves exhibited only a slightly better cytotoxicity effect on colon cancer cells as compared to fruit, despite their higher total phenolics, total flavonoids, and antioxidants. However, a nearly similar cytotoxicity effect as the fruit was exerted on liver hepatocellular cells. Many studies have reported *M. alba*’s multidirectional mechanism of action, which consists of eliminating reactive oxygen and nitrogen species and reducing the number of negative mutations and inflammation, while promoting apoptosis and activating the immune system. However, further investigations on in vivo studies of *M. alba* cytotoxicity are suggested to be conducted on more aggressive and metastatic cancers or late-stage cancers, thus giving a better chance for advanced-stage cancer patients.

## 4. Toxicity Study of *Morus alba*

The toxicity of *M. alba* leaves was previously determined in 4 weeks of oral administration of low (1%) and high (5%) doses to both male and female rats [102]. Throughout the observation period, no disturbances to growth or organ weight, nor any effects on the biochemical, hematologic, or pathological examinations were reported, indicating the consumption of *M. alba* leaves was safe. In an acute toxicity study, an intraperitoneal administration of *M. alba* leaf extract showed a median lethal dose (LD_50_) of approximately 4 g/kg in mice and 5 g/kg in Winstar rats [103]. However, they found no significant toxicity from the same 5 g/kg leaf extract when orally administered to both subject groups. During the test, the only effect recorded was the depression of the central nervous and respiratory systems, which recovered within 15 to 30 min. In a subchronic toxicity study, 60 days of orally administered *M. alba* leaf extract (1, 2, and 3 g/kg daily) showed no significant effects on blood chemistry or hematologic values, as well as no significant histopathological abnormalities in the major organs of the mice. There were no deaths reported in any of the studies [103].

Throughout the 14 days of the trial, the behavioral signals and biomass of the mice were not affected by 300 and 2000 mg/kg of body weight of ethanolic *M. alba* leaves [87]. However, significant reduction of mean corpuscular volume (MCV) and mean corpuscular haemoglobin concentration (MCHC) occurred in the 2000 mg/kg-treated mice. The MCHC of the 300 mg/kg mice was also lower than the control group, indicating the activity of leaf compounds on erythrocytes, leading to lower cell production. Both mice groups showed a lower proportion of lymphocytes with an increase of segmented leukocytes. The 2000 mg/kg-treated mice showed significant alteration in alanine aminotransferase (ALT) and alkaline phosphatase enzymes. The liver cells’ instability signal of the Gamma-glutamyl transferase level (GGT) was the same in all groups, which indicated the nonhepatotoxic effect of *M. alba* leaves. Conversely, the 2000 mg/kg dose altered kidney and liver structure, whereas the 300 mg/kg only altered leukocyte proportion, with no toxicity or any irreversible cellular damages observed [87]. Nonetheless, compared to these results, the intraperitoneal injection of the same concentrations extracts was more damaging to the mice, as histological analysis showed significant alteration in the liver, kidney, and spleen [104]. Studies indicate that the ethanolic leaf extract at 2000 mg/k exerted low oral toxicity on mice. However, there were no deaths, so it is safe to use with caution. *M. alba* leaves could induce biochemical, haematological, and histopathological alterations.

An acute toxicity study on *M. alba* fruit showed a nonsignificant effect of 1000 mg/kg of polysaccharide intragastric administration on animal behavior. The results considered their respiratory distress, posture, emaciation, and mortality throughout the 1-week observational period [105]. A subchronic toxicity study of 90 days of *M. alba* fruit oral administration (0, 40, 200, and 1000 mg/kg) on Sprague Dawley rats reported no significant adverse reactions, with no influence on food and water intake, or on body mass gain [106]. No significant toxic impact was detected between the treated group and the control based on organ weight, biochemical values, and hematological and urine analysis [106]. No deaths were reported in any of the studies.

Furthermore, an in vivo neurobehavioral study of *M. alba* fruit (100, 300, 1000 mg/kg) showed no significant effect on the general health, metabolism, and growth of mice based on their alertness, body weight, daily food and water intake, and organ weight [107]. The low liver toxicity biomarkers, ALT and aspartate transaminase (AST), indicated a nondetrimental effect of *M. alba* fruit on liver. No indication of renal toxicity was observed, as the levels of renal-function biomarkers (blood urea nitrogen, creatinine, cholesterol, glucose, and albumin) were within normal ranges. The histological analysis confirmed that no morphological changes or internal injuries appeared, even at the highest oral dose of *M. alba* fruit (1000 mg/kg) throughout 28 days, indicating the safe consumption of *M. alba* fruit [107]. However, this study was not associated with neurochemical estimation. Therefore, to understand the precise mechanism of action, estimating the brain’s neurotransmitter levels is necessary, and could suggest the nontoxic effect of fruit extract on the normal growth of animals.

## 5. Functional Ingredients in Food Applications

*M. alba* leaves, fruit, and seeds are composed of different matrices, therefore varying their potential application in food industry. A substantial amount of nutrients was found in *M. alba* food products, including carbohydrates; protein; minerals like calcium, iron, and zinc; and vitamins. Yu et al. [33] found a high content of crude protein, total phenolic, and DNJ in *M. alba* leaves, indicating their health properties and potential as ingredients in the food industry. Recently, *M. alba* leaves have been used for herbal tea, especially in Asian countries [5]. The bitterness of this tea was found to be positively correlated with the content of TPC and TFC in the leaves and their radical-scavenging abilities [108].

The antioxidative properties of *M. alba* leaves also have demonstrated its food functionality in paratha [109]. Paratha with *M. alba* leaves added revealed an increase in the dough’s protein levels, fat, ash, polyphenol, DPPH, ABTS, and Fe^3+^-reducing and chelating capabilities in a dose-dependent manner. However, upon frying, a decrease in polyphenolic compounds (by 0.16–0.21%) and DPPH activity (by 22–34.6%) occurred due to polyphenol degradation caused by heating [110], while an increase in the activity of ABTS scavenging (by 1.4–6.6%), Fe^3+^ reduction (by 1.3–2.9%), and Fe^3+^ chelation (by 247.5–4906.3%) occurred due to Maillard-reaction products (MRPs) that allowed metal chelation to inhibit oxidation [111]. A higher cooking temperature with a shorter cooking time can have less impact on bound polyphenols and sugars than the contrary [112]. Further studies on commercial-range parathas, such as the frozen and the ready-to-cook forms, are more convenient alternatives, and the cooking method should be revised to limit the loss of mulberry’s functional activities.

Moreover, better pork quality was observed when 15% *M. alba* leaf powder (MLP) was added to the pig’s diet. The levels of backfat, shear force, lower cooking, and drip loss were decreased; and the content of inosinic acid, intramuscular fat value, pH, meat color value, and antioxidative capacity were increased [113,114]. In addition, 15% MLP-treated pigs showed higher mRNA levels of type I and IIa muscle fibers that were lower in shear, more sufficient in diameter, and more tender than the type IIb fiber [114]. However, the 15% MLP also caused adverse effects on the pig’s growth, as it reduced its average daily gain, feed efficiency, carcass weight, and dressing percentage.

On the contrary, the inclusion of 15% and 30% MLP in the diet of lambs maintained growth and carcass performance while increasing the total weight of their rumen and stomach. The 15% MLP supplementation showed to be the most promising concentration to maintain growth, intake, and carcass performance, and alleviate oxidative stress, improved blood metabolites, and improved quality of the longissimus lumborum muscle (redness) [115]. Despite the nonsignificant effect on the meat’s pH, the mRNA expressions of Cu/Zn SOD and GPx against lipid peroxidation were elevated, which implied MLP had an ability to enhance lamb meat’s redness, quality, and shelf life.

According to Zhang et al. [116], *M. alba* leaf extracts can prevent oxymyoglobin and metmyoglobin oxidation to maintain refrigerated beef’s color. In addition, leaf extract significantly reduced the values of peroxide and thiobarbituric acid reactive substances (TBARS), and increased the superoxide dismutase and glutathione peroxidase activities during beef storage, which suggested that mulberry leaves were able to reduce the lipid oxidation reaction. Nonetheless, *M. alba* leaves are a good supplement for ruminants and pigs, as well as a good natural antioxidant to preserve the color and increase the quality and shelf life of meat, which are important in food industries. Further research is needed to find a suitable MLP dietary level with a lower negative impact on pig growth, and a longer observation period is required to find the maximum capability of *M. alba* leaf extract on meat quality and preservation, to ensure the appropriate amount needed for the desired shelf life to prevent the food-processing industry from economic losses.

*M. alba* fruit, on the other hand, was found to be rich in volatile compounds with higher phenolic contents compared to other berries and mulberry variety [38,117]. The fruit is a good source of pectin (4.75–7%), which has a wide range of food-industry applications as an emulsifier, thickener, stabilizer, gelling agent, and a fat or sugar replacement in low-calorie foods [118]. Due to its remarkable gel-forming property, the authors utilized pectin to produce jellies, jams, and preservatives. They found that pectin promotes multiple biological activities, such as anti-inflammatory, antibacterial, antioxidant, and antitumour activities. Thus, pectin-containing *M. alba* fruit has a probable function as a food ingredient [89].

Currently, *M. alba* fruit is used in cooking, baking, and for dessert purposes due to its sweet taste and attractive bright color that is ideal for sherbets, jams, fruit tarts, pies, jellies, teas, wines, and cordials [19]. The utilization of fruit as a natural food colorant is derived from their cyanidin and delphinidin glycoside anthocyanins. They are reddish-purple to purple pigments that possess high FRAP and DPPH antioxidant activities [47,119]. Previously, Natić et al. [119] isolated 14 anthocyanins compounds with a total of 45.42–208.74 mg cyn-3-glu/100 g frozen weight, whereas a more recent study by Kim and Lee [47] obtained 16 types of anthocyanin with total value of 0.95–28.61 mg/g DW. Among them, cyanidin-3-*O*-glucoside and cyanidin-3-*O*-rutinoside were the two significant anthocyanins in *M. alba* fruit, possessing antioxidant anti-inflammatory properties.

In jam processing, the color quality of *M. alba* jam was influenced by storage temperature. The 5 °C-stored jam possessed better color qualities (L* = lightness, a* = green to redness, b* = blue to yellowness) than the 25 °C-stored jam, but the difference was not statistically significant. In addition, during the four months of storage, no significant influence of light conditions on the quality and fluctuation of total anthocyanins and phenolic contents was observed [120]. This showed that mulberry jam was stable during storage. The high antioxidant and color-enhancing properties positively recommended *M. alba* and its anthocyanins as a natural, functional food colorant, since it is safer to consume than synthetics and is capable of delivering enhanced color quality with value-added properties to the product. However, a study on this colorant’s stability in other foods such as jellies, puddings, or yogurts should be explored to reinforce the coloring efficiency. To our knowledge, no one has conducted further analysis of the antioxidant capacity of an *M. alba* anthocyanin-containing finished product, or of other biological benefits, despite the proanthocyanidin-significant antimicrobial effects in the gastrointestinal tract and inhibition of sugar-, protein-, and fat-uptake-related enzymes [121], which are valuable in products targeting concerned about calories or their body weight. Therefore, such studies on *M. alba* anthocyanins and their stabilization methods will determine their benefits as functional food ingredients.

Furthermore, the investigation of *M. alba* fruit’s polyphenolic compounds (MFPs) in dried minced pork slices (DMPS) revealed a dose-dependent antioxidative activity during processing and storage [122]. The MFPs effectively inhibited oxidation of muscle protein and oxidation-induced texture deterioration, and reduced the DMPS hardness during heating and storage. MFP also significantly inhibited aerobic bacteria growth on DMPS and masked the odor and flavor of pork. Interestingly, MFP-added samples during the storage and post-heating process revealed higher redness (a*) values as compared to the preparation-stage samples and the control group. Meanwhile, an increment not surpassing the control group occurred for yellowness (b*) and lightness (L*) [122]. Similar results were found in MFP-added Cantonese sausages during 28 days of room-temperature storage in Xiang et al. [123]. A* and L* elevations were suggested to be caused by anthocyanin degradation during heating and storage. Simultaneously, the rise of b* value was attributed to the oxidation of ferrous heme iron induced by lipid oxidized products. The samples’ hardness showed a good increment, owing to the polyphenols’ physicochemical modifications during storage. Moreover, MFPs at 1.0 g/kg provided protection against lipid- and protein oxidation-induced damage, based on the lower TBARS and carbonyl contents, yet at a higher level of sulfhydryl than sausages without MFPs. Aside from the effective control of volatile base nitrogen (TVB-N) and microbial stability, MFPs also significantly reduced residual nitrite, which indirectly reduced the production of carcinogenic nitrosamine, causing MFPs to be trusted in biosafety [123]. In summary, MFPs are a promising natural antioxidant with effective protection against TVB-N increment, lipids, and protein oxidation, which could also effectively regulate Cantonese sausages’ biosafety. While this study can be used as a preliminary reference for research on other meat-based products, due to its slight unfavorable color defect, further research using the antioxidant function for better sensory and quality properties should be prioritized.

A recent study added *M. alba* leaves and fruit respectively to liquefied and creamed rape honey to assess their effects on the enriched honey’s phenolic profile, antioxidant, and glycoside hydrolysis activities [124]. Rape honey is naturally deficient in polyphenols, but its antioxidant activity was enriched and diversified by 70- and 7-fold after the addition of *M. alba* leaf and fruit extracts, respectively. A similar increment also occurred in *M. alba*-enriched creamed rape honey. Furthermore, dose-dependent elevations of α-glucosidase and β-galactosidase were observed in leaf-enriched creamed honey. Meanwhile, in *M. alba*-fruit-enriched honey, a slight decrease in α-glucosidase and a non-dose-dependent β-galactosidase increase occurred. The addition of *M. alba*, especially the leaves, reduced about 50% of diastase, suggesting their inhibitory effect against carbohydrate-hydrolyzing enzymes [124]. This is advantageous, as it slows down the loss of the diastase enzyme in honey, which allows longer storage while still containing a major amount of beneficial enzymes.

The same compound enrichment effect was observed in *M. alba* (leaf extract + fruit) added to baked bread (MAB) studied by Kobus-Cisowska et al. [125]. They reported significantly high total phenolic acids, flavonols, and antiradical activities in bread after baking and after 30 days of frozen storage. The MAB also revealed substantially high isoquercetin, chlorogenic acids, and protocatechuic acid. Respective positive correlations between the sum of phenolic acid and the sum of flavonols with DPPH inactivation, ABTS, and reducing the power activities were reported. These high values show significant *M. alba* functionality in enhancing health benefits in foods for consumption. MAB also revealed higher sweet and insipid scent intensity, despite its grassier and bittersweet taste compared to the control bread’s sour and salty taste. MAB bitterness is due to *M. alba*’s polyphenols, such as vanillic acids and ferulic acids, that contribute to a bitter, bean-like taste. Meanwhile, the flavonol glycosides affect bitter and sour taste. Despite the differences, MAB received a similar level of scent desirability with higher taste desirability, expressed via sensory profiling compared to bread without *M. alba* enrichment. DPPH and ABTS activity, which expressed the continuous activation of the responsible antiradical compounds, was stable throughout 30 days of frozen storage in another study on MAB [125]. However, changes in the intensity of frozen MAB characteristics did occur; its sweet scent and grassy taste declined, yet its grassy scent and sweet taste intensified. From these data, *M. alba* leaf and fruit extracts could be used as natural food fortifiers in bread, as they enhanced the nutrition level, bioactive compounds, and antiradical activity without negatively influencing the bread’s sensory and microbiological qualities. Nevertheless, they should have conducted a prior analysis on the dough’s bioactive compounds and antiradical levels in this study in order to know the percentage of compound and antioxidant-activity loss after baking. Despite the positive results from various studies, these data are still far from enough to solidify a food-industry function. A broader food-variety study, concise shelf-life of products, and analysis of *M. alba* extracts’ effects, abilities, and best preservation of shelf-life are some aspects for further exploration.

Essential oils (EOs) are plant-based aromatic oily liquids usable in several industries, including the food industry, as functional flavoring substances due to their health benefits. They are also integrated into food packaging to increase food shelf-life due to their antimicrobial and antioxidative activities [126]. Despite EOs’ increasing popularity, there have only been a couple of studies conducted on *M. alba* EO compound constitution. Zhi-ming et al. [127] and Radulović et al. [128] found some volatile compounds in *M. alba* leaf EOs with noteworthy biological effects. These compounds mainly consist of terpenoids, phytol, heptacosane, hexahydrofamesyl acetone, hexadeconic acids, β-bisabolene, carotenoid derivatives, and geranyl acetone. These compounds are used as food additives and flavor enhancers. For example, phytol contains antioxidant, anti-inflammatory, and antinociceptive activities [121,129]. Hexadecanoic acid is famous for its anti-inflammatory [130] and cytotoxicity potentials [131], whereas β-bisabolene possesses a strong cancer cytotoxicity effect [132] and has a balsamic odor that led to its approval as a food additive. Heptacosane has been used as a nutritional supplement and sweetener to mend aftertaste [133]. Meanwhile, terpenoids are used for their medicinal, flavor-enhancing, and fragrance properties in various industries [134]. The same wide application occurred for carotenoid derivatives, which are also extensively utilized as a food colorant and provitamin, and as a fortifier to introduce its health benefits into food [135]. Nonetheless, no specific analysis of *M. alba* EOs or their compound potentials have been conducted before. Therefore, further studies of their biological properties and applications as a functional food additive are needed.

Leaf EOs aside, *M. alba*-fruit-derived oil revealed rich fatty-acid contents, such as linoleic acids (58.89% DW), palmitic acids (12.46% DW), oleic acids (11.87% DW), and stearic acids (5.67% DW) [136]. Moreover, *M. alba* seed oil revealed its high content of total phenolics, total flavonoids, tocopherols (1644.70–2012.27 mg/kg oil), and fatty acids such as linoleic acid (75.83–81.1%), palmitic acid (8.80–9.51%), oleic acid (5.64–10.38%), stearic acid (3.96–5.01%), linolenic acid (0.41–0.45%), and myristic acid (0.07%) [53,54,55]. The high amount of bioactive compounds in the seeds contributed to its high DPPH (62–72%), ABTS (0.026–0.072 mmol/g), and FRAP (0.15–0.68 mmol Fe^2+^/g) abilities, which were proven by the strong correlation between total phenolic content with DPPH and FRAP (r = 0.965 and r = 0.951, respectively) [54]. Moreover, the high flavor and volatile compounds in the leaf EOs could offer antioxidant [137], antifungal [138] and antibacterial [139] effects in medicine when utilized as a flavorer, fragrance enhancer, antimicrobial agent, and food preservative for pulses, grains, cereals, fruits, and vegetables in the food industry [140]. Meanwhile, *M. alba* seed oil could be a good source of tocopherol and essential fatty acids, especially linoleic acid, an omega-6 PUFA usable as a blood-vessel cleaner to decrease serum cholesterol and inhibit arterial thrombosis formation [141]. Therefore, the oil derived from *M. alba* leaves, fruit, and seeds contains many beneficial compounds and is potentially applicable in the food industry as a functional food ingredient. Nonetheless, one of the main complications in utilizing an oil is the quantitative or qualitative diversity of its compound content, which could cause variable biological efficacy [142]. EOs and oil carry a strong aroma, which might restrict their application due to the complex matrices and different interconnecting microenvironments of foods. It also requires a level of natural flavor complexes for adequate effectiveness that might exceed the organoleptic acceptable range, affecting the natural taste of food and human health [126,143]. The limited number of studies and data on *M. alba*-derived EOs and oils are far from enough to solidify *M. alba*’s potentiality in any industry. Therefore, further studies that isolate more of their compounds, and analyses of *M. alba*-derived EOs and oils for their nutrition, biological activities, potential applications, effectiveness, toxicity, and stability in the food system are implored to solidify their place as functional food ingredients.

### Microencapsulation of Morus alba

*M. alba*, with its biologically beneficial polyphenolic compounds, is a suitable functional food ingredient. Unfortunately, the poor stability and heat and light sensitivity of phenolic compounds are a widely known weakness [144]. There is evidence that the stability of *M. alba* polyphenols can be destroyed by environmental factors, including pH, temperature, and oxygen [145]. These downsides have limited their commercial applications, especially for hot-processed foods, as reported in Cheng et al. [146] and their previous studies. Many studies have investigated methods to stabilize and protect polyphenols from degradation. Microencapsulation is an effective technique to ameliorate the unstable physiochemical properties of a substance or compound, including their solubility and dispensability [147]. Xu et al. [145] stated that *M. alba* polyphenol (MP) stability in dried minced pork slices was significantly enhanced after gum Arabic microencapsulation with a core/wall ratio of 1:90. After 20 days of storage, the microencapsulated polyphenol (MMP) pork slices showed lower loss of total phenolic, flavonoid, and anthocyanin contents (15.10%, 16.87%, and 19.88%, respectively), with higher recovery (by 1.35-fold, 1.26-fold, and 1.16-fold, respectively). Moreover, both MP and MMP exhibited a synergistic inhibitory effect on protein and lipid oxidation, with MMP showing better oxidation and color stability. In addition, Cheng et al. [146] found 1% β-cyclodextrin MMP significantly improved the dried minced pork slices’ recovery of total phenolic, flavonoid, and anthocyanin contents as compared to the nonencapsulated product by 5.6%, 5.8%, and 18.6%, respectively. Furthermore, as compared to the control, the MP-added pork slices showed 1.03-fold higher FRAP and 1.18-fold higher ABTS▪+ scavenging ability, whereas the β-cyclodextrin MMP showed 1.2-fold and 3.58-fold increases, respectively. These positive effects were attributed to β-cyclodextrin’s protective ability on phenolic compounds. However, the degradation of certain compounds such as quercetin, cryptochlorogenic acid, and caffeic acid, were not effectively avoided, presumably due to the difference in structures, thermochemical stabilities, and reaction modes between the compounds and β-cyclodextrin.

The promising results for phenolic-compound recovery was strongly supported by the β-cyclodextrin microcapsule of MP, which showed optimal encapsulation efficiency at a core/wall ratio of 1:6 with ultrasound treatment at 450 W, 25 °C for 90 min. Under the optimized parameters, the processing stability of MMP, including the thermal, light, and storage stabilities, were significantly enhanced. Simultaneously, the encapsulation efficiencies of MMP for total polyphenol, flavonoid, and anthocyanin contents were higher than 97%, thus verifying the success of MP encapsulation in ameliorating the unstable properties of *M. alba*’s polyphenols [144]. Gum Arabic and β-cyclodextrin are some credible encapsulating agents for *M. alba* polyphenols to add higher nutritional value to food. The results created a solid base for further MMP studies on their natural-pigment and biopreservative abilities in other thermal-processed products when substituted for synthetics. However, detailed laboratory measurements with evaluations of color and sensory characteristics should be the focus of future studies. Moreover, the bioavailability of MMP and its synergistic inhibitory effect on protein and lipid oxidation in functional meat products must be further studied in larger-scale production to accommodate MMP integration at the commercial scale.

## 6. Industrial Scale-Up

The assimilation into industrial and commercial production involves a larger production size to accommodate market demand, thus the need for upscaling, which proceeds from the bench or laboratory scale to the pilot scale, and finally to the demonstration scale for commercial demand [148]. The bench or laboratory plant is an early-stage study that provides a sample’s successful assessment, clinical trials, and system parameters. This stage provides initial upscaling data and factors before following up with efficient pilot-plant production. However, the procedures and materials operated at a laboratory scale are usually not practical on a larger scale. The unsuitable scale-up factor, policies, and parameters often critically influence products’ values, properties, and volumes, leading to energy and economic losses [148]. The upscaling process requires deliberate time, efforts, experiences, in-depth research, and high capital before its successful implementation. These difficulties are the reasons for lower upscaling information than the same sample’s laboratory-scale study.

So far, only a handful of the attempted pilot-scale studies were successful, while most of them require more modification and trials. In Flaczyk et al. [149], the authors obtained about a 4% lower *M. alba* leaf-extraction yield than at the laboratory scale. The study also reported lower protein (by 13.61%), glucose (by 38.78%), phenolics (by 44.94%), phenolic acids (by 53.03%), and flavonols (by 11.36%), as well lower DPPH (by 35.72%) and ABTS (by 20.1%) activities. Nonetheless, at the pilot scale, they found higher concentrations of saccharose and galactose by 23.77% and 23.68%, respectively. These values were due to intermittent loss during pilot-scale processing, as well as the different extraction and concentration conditions between the two scales. Therefore, further analysis and determination of qualitative indicators are necessary for the optimization process.

Gang et al. [150] obtained a polysaccharides’ optimal extraction preceding data focusing on *M. alba* leaves. This study concluded the optimized temperature, sample-to-solvent ratio, and extraction time via a Box–Behnken response surface design (BBD) coupled with response surface methodology (RSM). As such, extraction at 76.7 °C with a 1: 20.6 sample-to-solvent ratio, extracted for 1.87 h, was reported to produce the highest polysaccharides (105.57 mg/g), consistent with their predicted value (106.64 mg/g). A recent pilot-plant study reported the optimal extraction condition for DNJ from *M. alba* leaves [151]. The RSM exploration showed the best DNJ extraction value (31.62 mg/g) at 79.2 °C with a 1:9.82 sample-to-solvent ratio for 1.46 h. The value was higher than for the small-scale extraction process. These studies showed that extraction-parameter optimization is crucial in obtaining the best outcome value, and that RSM or BBD coupled with RSM were reliable methods for optimization of upscaled extraction. The obtained data on optimized extraction of *M. alba* leaf polysaccharides and DNJ can be used as a foundation for industrial applications of *M. alba*.

Sample-processing parameters aside, the equipment and types of machinery used at the pilot scale also affect the process and outcome. Komaikul et al. [152] found that both stilbenoid and mulberroside A from an *M. alba* cell culture were affected by the usage of different types of bioreactors. The study revealed that round-bottom bioreactors could produce three times higher biomass than the flat-bottom bioreactor, though no significant difference in mulberroside A production was found. However, significantly higher mulberroside A was obtained from shake-flask cultured cells without an air-driven system compared to air-driven bioreactors. Meanwhile, the stilbenoid production preferred smaller air-driven bioreactors (1 L) with low aeration rates. In sum, the production of mulberroside A and stilbenoid in *M. alba* cell cultures could be affected by factors such as biomass circulation, aeration, and endogenous enzymatic hydrolysis. Therefore, a study focusing on target-compound stabilization and reduction of aeration-induced negative effects would be a valuable approach for *M. alba* at the pilot-plant scale. More studies and optimization of *M. alba* pilot-plant processing should include factors of formulae standardization, chemical equilibrium, product-quality maintenance, thermodynamics, processing equipment, energy consumption, etc., to ensure *M. alba*’s successful application at the industrial level.

## 7. Conclusions and Future Perspectives

Taken together, *M. alba* leaves and fruit have a high content of bioactive compounds such as phenolic acids, flavonoids, flavonols, anthocyanins, macronutrients, vitamins, and volatile aromatic compounds. These compounds contribute significantly to the properties of *M. alba* in preventing and treating illnesses such as oxidative stress, diabetes, hyperlipidaemia, neurological disorders, microbial infections, and cancer. Numerous studies have been conducted on *M. alba* fruits and leaves, especially to determine their bioactive compounds, pharmaceutical potentials, and toxicity effects. However, limited studies have been done on *M. alba* seeds. Therefore, it is essential to include knowledge about the seeds so that the full potential of *M. alba* can be achieved.

There are significant knowledge gaps between *M. alba* leaves and fruit regarding their working mechanisms, bioavailability, and biochemistry in the human body. Moreover, the multiple phytochemicals and bioactive compounds contained in different *M. alba* cultivars, maturity stages, extraction conditions, and methodologies have produced heterogeneous data. This has led to difficulty in evaluating and standardizing their activities. For that reason, more suggestive scale trials with well-designed and uniform parameters will provide a substantial input in obtaining homogenous data, which would benefit further comprehension of the in vivo effect on human health of *M. alba* leaves and fruit, including their bioavailability and biological effect against enzymes, pH, and especially gut microbiota.

There is limited information available on the application of *M. alba* in the food industry as a functional food ingredient, despite its current presence in commercial products. Studies have found that *M. alba* has promising prospects for functional food applications. Its flavonoids and phenolic acids can act as flavorants, food fortificants, antioxidants, preservatives, and antimicrobial agents against bacteria and fungi. In contrast, its anthocyanins could be used as natural antioxidative food colorants to substitute for synthetic colorant usage. With the wide variations of food, more studies on a broader range of foods such as frozen, ready-to-eat, or processed foods are necessary to understand the reactions and impact of *M. alba* on other food ingredients, as the food system itself is very complex. In the vast food-application potential, research emphasizing the effect of processing and storing on the content and stability of bioactive compounds in *M. alba*-based foodstuffs is needed for a better understanding of the bioactive compounds’ role and interaction in the food matrix to enhance food quality and properties.

Additionally, in regard to the degradation of heat-sensitive bioactive compounds, microencapsulation techniques have been proven to naturally protect and significantly enhance *M. alba* polyphenols’ stability and properties. This result could establish a factual basis for further study of their utilization in heat-processed products and better industrial-processing performance, thus expanding the application of *M. alba* in various food products. However, integration into the industry requires scaling up from the laboratory scale to the pilot plant, and subsequently to the industrial plant, to produce a larger production scale to ensure demand can be met. Regrettably, studies on the pilot scale-up process for *M. alba* leaves and fruit are still very lacking. This is possibly due to the difficulty of scale-up implementation of bioprocessing. It requires deliberate time, efforts, experiences, in-depth research, and high capital. Unsuitable scale-up factors, procedures, and parameters would critically influence product values, properties, and volumes, leading to undesirable energy and economic losses to a company. In conclusion, *M. alba* indeed possesses beneficial biological properties and is suitable to be included as a functional food ingredient. Nevertheless, the exploitation of *M. alba*’s functionality and properties within the industrial field are still scarcely explored.

## Figures and Tables

**Table 1:** Macronutrients and vitamins in *M. alba*.

	Nutrients	Carbohydrate	Protein	Fats	Fibre	Ascorbic Acid	β-Carotene	References
Sample								
*M. alba* leaves		28.8%	27.1%	1.9%	26.5%	-	-	[17]
		9.7–29.6%	15.3–30.9%	2.1–4.9%	27.6–36.7%	100–200 mg/100 g	8.4–13.1 mg/100 g	[13]
		9.7–39.7%	14.0–34.2%	3.5–8.1%	5.4–38.4%	-	-	[18]
*M. alba* fruit		-	-	1.10%	-	22.4 mg/100 mL	-	[1]
		-	10.2– 13.3%	-	-	-	-	[6]
		-	12.98%	-	8.32%	351 mg/g	13.7 mg/100 g	[15]
		71%	13–15%	2–3.5%	12–14%	-	-	[19]
		-	1.55 g/100 g	0.48 g/100 g	1.47 g/100 g	15.2 g/100 g	-	[20]

**Table 2:** Mineral contents in *M. alba*.

	Minerals	Ca	Zn	Fe	Mg	P	K	Mn	Na	References
Sample										
*M. alba* leaves		14.9 mg/g	2.2 mg/100 g	27.1 mg/100 g	5.3 mg/g	3.7 mg/g	12.4 mg/g	-	58.62 mg/100 g	[17]
		1.0–1.6%	-	-	0.4–0.7%	0.2–0.3%	1.3–1.9%	-	-	[21]
		11.2–27.6 mg/g	-	-	2.2–3.4 mg/g	3.8–7.02 mg/g	16.1–18.6 mg/100 g	-	-	[22]
*M. alba* fruit		1.52 mg/g	2.8 mg/100 g	4.2 mg/100 g	1.06 mg/g	2.47 mg/g	16.68 mg/g	3.8 mg/100 g	60 mg/100 g	[1]
		0.2–0.4 g/100 g	14.9–19.6 mg/kg	28.2–46.7 mg/kg	0.1–0.2 g/100 g	0.2–0.3 g/100 g	1.6–2.1 g/100 g	12.3–19.4 mg/kg	0.01 g/100 g	[6]
		2.73 mg/g	1.8 mg/100 g	6.3 mg/100 g	1.82 mg/g	1.98 mg/g	9.07 mg/g	0.8 mg/100 g	82.8 mg/100 g	[23]
		5.76 mg/g	50.20 mg/100 g	73 mg/100 g	2.4 mg/g	-	17.31 mg/g	-	2.8 mg/g	[20]

**Table 3:** Major phytochemical classes in *M. alba* leaves.

Phytochemical Class	Amount in Leaves	References
Flavonoids	57.8%	[32]
	21.36–56.41 mg RE/g DW	[33]
	20.4–187.23 mg QUE/g	[34]
	3.66–6.11 mg/g DW	[5]
Benzofurans	17.9%	[32]
Phenolic acids	10.7%	[32]
	0.84–1.07 mg/g DW	[25]
	6.78–8.48 mg/g DW	[5]
Alkaloids	6.4%	[32]
	0.680–6.909 mg/g	[35]
	1359 mg/kg	[36]
Coumarins	3.6%	[32]
Chalcones	2.9%	[32]
Stilbenes	0.7%	[32]
	188.57 mg/100 g DW	[37]

**Table 4:** Major phytochemical classes of *M. alba* fruit.

Phytochemical Class	Amount in Fruit	References
Phenolic acids	0.013–0.57 mg CAE/g DW	[30]
	0.90–2.18 mg/g DW	[46]
	1.17–3.62 mg/g DW	[6]
	0.62–0.84 mg/g DW	[25]
Flavonoids	0.026–0.607 mg QE/g	[30]
	0.553–2.83 mg/g DW	[46]
	3.66–6.11 mg/g DW	[6]
Alkaloids	660 mg/100 FW	[20]
	1047 mg/kg	[36]
Anthocyanins	0.95–28.61 mg/g DW	[47]
	6.52 mg/100 g DW	[24]
Sterol	28.07%	[45]
Stilbenes	609.15 mg/100 g DW	[37]

**Table 5:** Antioxidative properties of *M. alba* from various studies.

*M. alba* Materials	Extraction Solvent	Assay	Result	References
Leaves	Ethanol, Petroleum ether, Ethyl acetate, and *n*-butanol.	DPPH	IC_50_ = 145–2070 mg/mL	[56]
		ABTS	IC_50_ = 32.73–642.33 mg/mL	
		FRAP	EC_50_ = 868.67–2429.33 mg/mL	
Leaves	Methanol	DPPH	33.22–56.37 μmol TE/g DW	[33]
		FRAP	91.62–149.15 μmol AAE/g DW	
		ABTS	51.28–70.84 μmol TE/g DW	
Leaf oligopeptides	Liquid nitrogen	DPPH	322.7–876.9 μg/mL	[58]
		ABTS	141.3–259.6 μg/mL	
		Nitric oxide scavenging	5.11–176.4 μg/mL	
		Metal chelation	169.6–328.6 μg/mL	
		Anti-lipid peroxidation	202.3–315.5 μg/mL	
Leaf Moracin N, Resveratrol, and Quercetin	Ethanol	Oxygen radical absorption capacity	1.55–10.86 μmol TE/μmol	[61]
		DPPH	IC_50_ = 40.00–285.54 μM	
		Cellular antioxidant activity	No PBS wash: 6.68–22.51 μmol QE/100 μmol	
Fruit	Ethanol	DPPH	IC_50_ = 0.518 mg/mL	[34]
		FRAP	0.522–0.685	
		Ferrous ion chelation	72.6%	
		Lipid peroxidation	39–45.51%	
Fruit	Ethanol, Hexane, Chloroform, Ethyl acetate and n-Butanol	DPPH	EC_50_ = 71.12–623.86 mg/L	[39]
		Superoxide anion radical-scavenging	EC_50_ = 82.37–921.83 mg/L	
Fruit	Ethanol, N-hexane, Dichloromethane, and Ethyl acetate	DPPH	35.43–66.6%	[45]
Fruit polysaccharides	Ethanol	Oxygen radical absorption capacity	1117.3–2159.8 μmol TE/g	[62]
		Rapid peroxyl radical scavenging capacity	75.23–461.32 μmol Vit C/g	
		Cellular antioxidant activity	No PBS wash: 19.64–66.41 μmol QE/100 g	
			PBS wash: 3.13–7.66 μmol QE/10 g	
Seed polyphenols	Methanol	DPPH	IC_50_ = 20.2–48.2 μM	[48]

## Data Availability

No new data were created or analyzed in this study. Data sharing is not applicable to this article.
